# Heterogeneous graph transformer and diffusion model for disease diagnosis

**DOI:** 10.1038/s41598-025-32220-6

**Published:** 2025-12-17

**Authors:** Xiaodong Zhu, Dan Yang, Yang Liu

**Affiliations:** School of Computer Science and Software Engineering, University of Science and Technology Liaoning, Anshan, 114051 China

**Keywords:** Heterogeneous graph neural networks, Graph transformer, Diffusion model, Disease diagnosis, Computational biology and bioinformatics, Engineering, Mathematics and computing

## Abstract

With the continuous development of Electronic Health Record (EHRs), medical heterogeneous data have become increasingly abundant, containing diverse types of entities and complex semantic relationships that provide essential support for disease diagnosis. However, conventional heterogeneous graph neural networks struggle to distinguish semantic differences among multi-type nodes and k-hop neighbors, often leading to semantic confusion and vulnerability to noise, which limits their classification performance and generalization capability. To tackle these challenges, we present a novel framework named Heterogeneous Graph Transformer and Diffusion mechanism for Disease Diagnosis (TD4DD), which captures multi-scale semantic dependencies across k-hop neighborhoods, while the diffusion module performs latent-space denoising to alleviate noise interference. Specifically, a k-hop hierarchical Transformer is introduced to capture multi-scale dependencies across different hop layers, enabling the differentiation of fine-grained semantic variations among neighbors at various distances. Additionally, a diffusion module is designed to handle noise in the data by performing denoising in the latent space through auxiliary subgraphs constructed using different meta-paths, thereby generating more discriminative node representations. Finally, the model fuses structural information with denoised embeddings to accomplish disease classification. Experiments conducted on two real-world clinical datasets, MIMIC-III(7,000 patients, 5 disease categories) and MIMIC-IV(8,331 patients, 6 disease categories), demonstrate that TD4DD consistently outperforms existing baseline methods in terms of both Micro-F1 and Macro-F1 scores, showing strong generalization ability. On MIMIC-III, TD4DD achieves a Micro-F1 of 88.29 and a Macro-F1 of 86.11, while on MIMIC-IV, it reaches 83.60 and 83.94, respectively. Furthermore, ablation studies and t-SNE visualizations further validate the effectiveness of each module and the distinguishing capability of the learned embeddings.

## Introduction

With the rapid development of medical informatics and big data, EHRs^1^ have accumulated massive heterogeneous clinical data involving entities such as patients, diseases, symptoms, medications, and procedures, along with complex interrelations. However, inaccurate diagnosis stemming from these data can lead to severe clinical outcomes, including delayed treatment and ineffective resource allocation. By abstracting these into a network structure, one can construct Heterogeneous Information Networks (HINs)^2,3^, which capture rich semantics and provide a strong foundation for precise disease diagnosis.

Graph Neural Networks (GNNs)^4^ have shown strong modeling capabilities in structured data, but their standard formulations for homogeneous graphs limit their applicability in heterogeneous medical networks. Existing heterogeneous GNNs^5,6^ often rely on meta-paths or simple neighbor aggregation to handle heterogeneity. However, when applied to real-world disease diagnosis tasks^7–9^, these models still face two major challenges.

First, EHRs often contain missing, redundant, or irrelevant information, which negatively impacts representation learning and prediction accuracy. Second, existing models struggle to capture complex semantics in medical HINs. HAN^10^ performs coarse-grained neighbor aggregation and fails to distinguish distance-sensitive semantics, leading to semantic confusion. Furthermore, many methods indiscriminately aggregate features across different node types, ignoring type-specific meanings and resulting in type-mixing. These limitations not only reduce classification accuracy but also pose potential risks for disease diagnosis in clinical settings. Semantic confusion in medication records may obscure important distinctions between primary and secondary drugs, while noise and missing data can lead to unstable or unreliable diagnostic predictions.

To address these clinical challenges, we propose a disease diagnosis framework named TD4DD, which integrates multi-level Transformer encoding and diffusion-based denoising on medical heterogeneous graphs, ultimately generating more reliable representations for diagnosis. The key idea is to hierarchically model both distance- and type-aware semantics using a Transformer structure, while leveraging diffusion mechanisms in the latent space to effectively suppress noise and extract task-relevant information. The main contributions are summarized as follows:

1. To alleviate semantic confusion caused by indistinguishable neighbor distances in existing methods, we propose a k-hop Transformer that model’s node representations obtained via meta-path-based sampling. A hierarchical Transformer is further designed to fuse semantics across hop levels. This module employs k-hop and hierarchical attention mechanisms to distinguish between 1-hop, 2-hop, and higher-order neighbor, enabling precise modeling of multi-scale relationships among diseases, symptoms, and treatments.
2. To address the issue of noisy data in medical HINs, we introduce a diffusion model into medical heterogeneous graph learning. Based on the meta-paths P-D-P and P-D-P-O-P, we construct auxiliary subgraphs and integrate them into the diffusion process, transforming noisy inputs into clean, task-specific embeddings and thereby enhancing representation robustness.
3. We propose the complete TD4DD framework and evaluate its performance on two real-world datasets, MIMIC-III and MIMIC-IV. Experimental results show that TD4DD consistently outperforms existing advanced baselines in disease diagnosis tasks, demonstrating its effectiveness and practical applicability.

## Related work

### Heterogeneous graph neural networks

Heterogeneous Graph Neural Networks are specifically designed for modeling HINs consisting of multiple types of nodes and edges. These methods aim to learn low-dimensional node embeddings by aggregating neighborhood information. Early works such as Metapath2vec^11^ utilizes predefined meta-paths to guide embedding learning. However, their performance heavily relies on the selection of appropriate meta-paths, and their generalization capability is limited when the meta-paths are numerous or undefined. To overcome the limitations associated with meta-path-based methods, HAN introduces node-level and semantic-level attention mechanisms. Specifically, node-level attention aggregates features from neighbors of the same type, while semantic-level attention assigns weights across different relation types. Nevertheless, existing neighbor aggregation methods face challenges of over-smoothing when aggregating k-hop neighbors and struggle to distinguish subtle semantic differences from neighbors at various distances, potentially resulting in semantic confusion.

While prior heterogeneous graph methods have advanced semantic understanding, their technical assumptions limit their applicability in noisy and sparsely connected medical graphs. HSGNN^12^ constructs similarity subgraphs via meta-paths and fuses them with trainable weights, effectively capturing hierarchical structure but lacking mechanisms to model hop-wise semantic variations within each meta-path, which are essential for distinguishing the diagnostic relevance of direct versus indirect clinical relations. HeCo^13^ proposes a cross-view contrastive paradigm between network schema and meta-path views, but it primarily enforces global representation alignment and cannot capture multi-scale dependencies through explicit hierarchical attention. MAGNN^14^ encodes intermediate nodes along meta-paths to enrich semantic aggregation, yet it remains a purely discriminative model and lacks a generative denoising process to mitigate the noise and sparsity inherent in EHR data.

In contrast, TD4DD bridges these gaps by introducing a k-hop hierarchical Transformer that explicitly captures semantic dynamics across different hop distances within a meta-path, and a diffusion-based latent denoiser that corrects embedding distortions caused by clinical noise. This integrated design enables fine-grained semantic disambiguation and robust representation learning under imperfect real-world medical graph conditions—an aspect largely overlooked in prior frameworks.

### Graph transformer models

Transformer models^15^, owing to their powerful self-attention mechanism, have shown remarkable performance in natural language processing and have been adapted to graph-structured data. In the clinical domain, Transformer-based models such as BEHRT^16^ and Med-BERT^17^ have demonstrated the capability of learning rich contextual dependencies from longitudinal EHR sequences, achieving strong performance in disease prediction tasks. However, these models cannot capture the complex relational structure among patients, medications, and procedures that naturally form heterogeneous graphs.

To overcome these limitations, Graph Transformers were introduced to extend self-attention to relational data. Graph Transformer^18^ replaces message passing in GNNs with self-attention, directly modeling long-range dependencies and mitigating over-smoothing. Early variants mainly targeted homogeneous graphs; for example, Graphormer^19^ incorporates structural encoding to enhance graph-aware modeling. However, their direct application to heterogeneous graphs remains limited, as they often neglect node and edge heterogeneity and lack explicit meta-path-based semantic modeling. FastGTN^20^ captures high-order semantic adjacency relationships through graph transformations, while SlotGAT^21^ introduces a slot mechanism to mitigate global information loss—though its static nature limits adaptability to temporal semantics in complex diagnoses. To address semantic confusion, HHGT^22^ employs hierarchical attention to distinguish neighbor semantics across distances, whereas most Graph Transformers still treat all neighbors equally, causing subtle but crucial semantic differences to vanish. Compared with traditional attention models such as GAT^23^ and GATv2^24^, Graph Transformers leverage global attention to associate distant heterogeneous nodes effectively. Nonetheless, they continue to face computational challenges when applied to large-scale medical graphs.

To address these issues, our k-hop hierarchical Transformer is designed to operate within each meta-path, capturing fine-grained semantic variations across hop distances and providing precise, clinically meaningful semantic representations under heterogeneous EHR settings.

### Diffusion and generative models for graphs

Denoising diffusion probabilistic model DDPM^25^ learn data distributions by simulating a forward noise-adding phase and a reverse denoising phase, and have recently been extended from vision and speech to graphs. In graph representation learning the denoising step provides cleaner, more robust node embeddings. DiffGraph^26^ adapts this paradigm to heterogeneous graphs, treating diffusion as a cross-view denoising strategy. During the forward phase it gradually diffuses target-graph embeddings into auxiliary views, and during the reverse phase it recovers refined target embeddings, thereby suppressing view-specific noise. Although effective, DiffGraph still struggles to capture the complex, high-order noise patterns that characterize electronic medical record graphs, suggesting the need for more flexible diffusion mechanisms in such settings. To address this, our diffusion module leverages multiple auxiliary subgraphs derived from distinct meta-paths, performing cross-view denoising to enhance robustness and maintain semantic consistency across different clinical relations.

### Disease diagnosis method based on K-hop hierarchical transformer and diffusion module

**Fig. 1:**
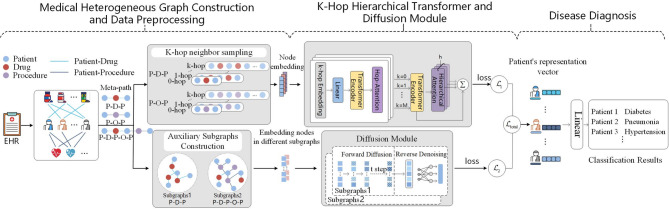
The overall architecture of TD4DD.

As illustrated in Fig. 1, we propose a disease diagnosis framework based on a k-hop hierarchical Transformer and a diffusion-enhanced heterogeneous graph neural network. The model is designed to capture multi-scale semantic dependencies and denoise patient representations by integrating structural information across meta-paths and auxiliary subgraphs.

### Medical heterogeneous graph construction and data preprocessing

We model the EHRs as a heterogeneous graph ${{\mathcal{{G}}}}\mathcal{=(}{{\mathcal{V}}},{\rm E},{{\mathcal{N}}}{{\mathcal{M}}}\mathcal{)}$, Here, ${{\mathcal{V}}}$ is the set of nodes such as patients (P), drugs (D), and procedures (O), and E is the set of directed edges such as P-D, D-P, P-O, and O-P. ${{\mathcal{N}}}$ and ${{\mathcal{M}}}$ denote the sets of node types and edge types, respectively. Each node ${{v}_{i}}$ in ${{\mathcal{V}}}$ is associated with a feature vector. For each relation type (P-D, D-P, P-O, O-P), we construct a corresponding adjacency matrix denote the number of source and destination node types, respectively. Each matrix is built from the observed edges in the EHR data, with entries set to 1 if a connection exists and 0 otherwise. Meta-path based adjacency matrices (e.g., for P-D-P) are computed by multiplying the corresponding relation matrices and applying binary normalization. To further capture the rich semantics embedded in indirect relationships, we define meta-paths as sequences of alternating node and edge types: $\mathcal{\mathcal{P} }:{{A}_1}\xrightarrow{{{{R}_1}}}{{A}_2}\xrightarrow{{{{R}_2}}}...\xrightarrow{{{{R}_l}}}{{A}_{l+1}}$. Here, ${{A}_{i}} \in \mathcal{\mathcal{N}}{,}{{R}_i} \in \mathcal{\mathcal{M}}$. These meta-paths serve as semantic templates, enabling the discovery of latent associations between patients and other clinical entities in the medical graph. We use three meta-paths: P-D-P, P-O-P, and the longer P-D-P-O-P, capturing increasingly complex interactions among clinical entities. Furthermore, for any patient node v, we define its k-hop neighborhoods as:

1 $${\mathcal{\mathcal{N}}_{k}}{(v)=}{{\{ }}{u} \in \mathcal{\mathcal{V}}\left| {{\mathrm{dist(}}{v,u}{\mathrm{)}}{=k}} \right.{\mathrm{\} ,}}$$

**Fig. 2:**
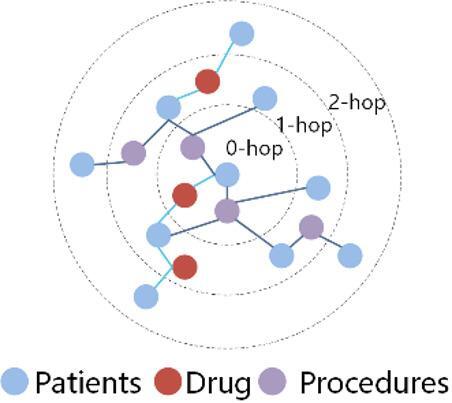
K-hop neighbor.

Here, dist(v, u) denotes the shortest path length between nodes v and u. These hop-wise neighborhoods enable fine-grained semantic encoding, as depicting in Fig. 2.

Neighbor sampling is performed along P-D-P and P-O-P up to k = 4 hops: each patient node expands only to drug or procedures nodes, while drug and procedures nodes expand exclusively to patients. The hop-wise feature sequence $\{ h_{v}^{{(k)}}\} _{{k=1}}^{4}$ produced for every patient node v is forwarded to a hierarchical Transformer that learns multi-scale structural representations. The maximum hop number k determines the range of information propagation in the graph and the amount of contextual knowledge the model can capture. We set k = 4 to obtain a balanced representation, and subsequent experiments verify the effectiveness of this choice.

To enhance robustness against noisy clinical records, we further construct two auxiliary subgraphs. The first, ${\mathcal{\mathcal{G}}^1}$, links two patients if they share at least one drug along the P-D-P path; the second, ${\mathcal{\mathcal{G}}^2}$, connects patients sharing either a drug or a procedure under P-D-P-O-P. For each subgraph, we construct its feature matrix ${X^{(i)}} \in {R^{N \times d}}$ and adjacency matrix ${A^{(i)}} \in {R^{N \times N}}$, which are then passed through the shallow GCN encoder ${{f}_0}$ to obtain the initial embeddings:

2 $$h_{0}^{{(v)}}={{f}_0}({X^{(v)}},{A^{(v)}}),$$

The two embeddings ${h^{(1)}}$ and ${h^{(2)}}$ are then used as multi-view inputs to the diffusion module, allowing it to enforce representation consistency and noise robustness across different semantic paths.

The proposed framework integrates hierarchical hop-level structural information with denoised multi-view embeddings, generating patient representations that maintain strong discriminative capability across multiple structural scales and exhibit robustness to clinical noise, thereby laying the foundation for the downstream diagnostic task.

### K-hop hierarchical transformer

To capture multi-level semantics in medical heterogeneous graphs, we design a k-hop hierarchical Transformer guided by neighbor sampling. For each patient node, neighbors are sampled from 1st to k-th hop and grouped by hop count to form structured sequences.

1. K-Hop Transformer Aggregation

To encode fine-grained semantics, a k-hop Transformer is applied to each hop-specific neighbor set of the target patient node. Neighbors from hop 1 to k are sampled and grouped by hop count into feature sequences, each processed by an independent Transformer to capture intra-hop contextual dependencies and generate hop-level representations. For each patient node, we sample a fixed number of neighbors (32) at each hop from 1 to k. The sampling follows the meta-path templates (P-D-P and P-O-P) to maintain semantic consistency. During training, we use a mini-batch size of 256 patient nodes. For each batch, we dynamically construct the k-hop subgraphs for all sampled patients, enabling efficient GPU memory usage and scalable training.

To integrate these encoded neighbor features into the target node representation, the model adopts an attention-based weighted aggregation. Specifically, it computes attention weights between the target node embedding ${h_v}$ and each encoded neighbor representation $h_{u}^{{(k - hop)}}$ using:

3 $$\alpha _{{hop}}^{{(k)}}(v,u)={Softmax}\left( {\frac{{({{h}_v}{{\mathrm{W}}_Q}){{({h}_{u}^{{(k - hop)}}{{\mathrm{W}}_K})}^T}}}{{\sqrt {{{\mathrm{d}}_h}} }}} \right),$$

where ${{\mathrm{W}}_{Q}}{\mathrm{,}}{{\mathrm{W}}_{K}}\,{{\mathrm{W}}_{V}}$ are learnable projection matrices and ${d_h}$ is the hidden dimension. The aggregated representation ${\mathrm{z}}_{v}^{{(k)}}$ for the k-hop is then computed as:

4 $${\mathrm{z}}_{v}^{{(k)}}=\sum\limits_{{u \in {\mathcal{\mathcal{N}}_k}(v)}} {a_{{hop}}^{{(k)}}(v,u)(h_{u}^{{(k - hop)}}{{\mathrm{W}}_V})} {\mathrm{,}}$$

where ${\mathcal{\mathcal{N}}_{k}}{\mathrm{(}}{v}{\mathrm{)}}$ denotes the set of k-hop neighbors of node v. This aggregation strategy enables the model to selectively focus on informative context within each hop and effectively encode k-hop neighborhood semantics, thereby improving the expressiveness of patient representations for downstream disease diagnoses.

2. Hierarchical Transformer Aggregation

After obtaining the aggregated representations from each hop level $\{ {\mathrm{z}}_{v}^{{(k)}}\} _{{k=1}}^{{K}}$, it is important to note that semantic information across hops is not independent—complex dependencies may exist between different hop layers. To capture such cross-hop interactions, these representations are concatenated into a hop-level sequence of length K, which serves as the input to the hierarchical Transformer.

The hierarchical Transformer incorporates multi-head self-attention and feed-forward layers to model global dependencies among hop-level features. After processing, it outputs a transformed sequence $\{ {\mathrm{z}}_{v}^{{(k)}}\} _{{k=1}}^{{K}}$, where each ${\mathrm{z}}_{v}^{{(k)}} \in {R^{{d_h}}}$ encodes the contextualized representation of the k-hop after intra-hop interaction.

To integrate these representations into a unified vector for the target node v, a hierarchical attention mechanism is introduced. This mechanism computes the relevance between the central node embedding ${h_v}$ and each ${\mathrm{z}}_{v}^{{(k)}}$, assigning dynamic weights to different hops based on similarity:

5 $${\beta _k}={Softmax}\left( {\frac{{({{h}_{v}}{{\mathrm{W}}_{Q^{\prime}}}){{({\mathrm{z}}_{{v}}^{{{(k)}}}{{\mathrm{W}}_{{K^{\prime}}}}{)}}^{T}}}}{{\sqrt {{d_h}} }}} \right),$$

The intermediate fused representation ${{h^{\prime}}_v}$ is then computed as:

6 $${{h^{\prime}}_v}{=}{{h}_{v}}{+}\sum\limits_{{{k=}{\mathrm{1}}}}^{{\mathrm{4}}} {{{{\upbeta}}_{k}} \cdot {\mathrm{(z}}_{{v}}^{{{(k)}}}{{\mathrm{W}}_{{V^{\prime}}}}{\mathrm{)}}} ,$$

where ${{\mathrm{W}}_{{Q'}}}{\mathrm{,}}{{\mathrm{W}}_{{K'}}}{\mathrm{,}}{{\mathrm{W}}_{{V'}}}$ are learnable projection matrices. To further enhance the expressive capacity, a multi-head attention mechanism is introduced to dynamically fuse representations across different hop levels. Let ${{h^{\prime}}_v}^{{(m)}}$ denote the output of the m-th attention head; the final node embedding is obtained by concatenating all head outputs followed by a linear projection:

7 $$h_{{v}}^{{HHGAT}}={W_{out}}\left\| {_{{m=1}}^{H}} \right.{{h^{\prime}}_v}^{{(m)}},$$

where H is the number of attention heads, || denotes concatenation, and ${W_{out}} \in {R^{d \times (H \cdot {d_h})}}$ is a learnable projection matrix that maps the concatenated output to the final embedding dimension d. This hierarchical Transformer aggregation enables the model to comprehensively capture multi-scale semantics within the k-hop neighborhood and dynamically assign importance across hop levels, resulting in semantically rich and well-separated of patient embeddings for downstream disease diagnosis.

### Diffusion module

To enhance the robustness and distinguishability of patient embeddings in medical heterogeneous graphs, we introduce a diffusion module that performs denoising in the latent space guided by auxiliary subgraphs derived from meta-paths P-D-P and P-D-P-O-P. A shallow GCN first generates initial embeddings from each subgraph, which are then corrupted via a forward diffusion process by progressively injecting Gaussian noise. The reverse process, parameterized by a graph-aware neural network, learns to recover clean embeddings by predicting the added noise, minimizing a diffusion loss ${\mathcal{\mathcal{L}}_{{\mathrm{diff}}}}$.

We begin by constructing two auxiliary subgraphs ${\mathcal{\mathcal{G}}^1}$ and ${\mathcal{\mathcal{G}}^2}$ based on the meta-paths P-D-P and P-D-P-O-P, respectively. For each subgraph ${\mathcal{\mathcal{G}}^{({i})}}({i} \in \{ 1,2\} )$, we extract its adjacency matrix ${A^{(i)}} \in {R^{N \times N}}$ and feature matrix ${X^{(i)}} \in {R^{N \times {d_x}}}$, where N is the number of patient nodes and ${{d}_{x}}$ is the original feature dimension. A shallow GCN is then used to produce the initial node embeddings:

8 $$h_{0}^{{(i)}}={\mathrm{GCN}}({X^{(i)}},{A^{(i)}}),$$

where GCN(·) denotes a graph convolution procedures and ${h^{(i)}}=[h_{1}^{{(i)}},h_{2}^{{(i)}},...,h_{t}^{{(i)}}] \in {R^{N \times d}}$ are the low-dimensional embedding used in the diffusion process.

The diffusion module consists of a forward diffusion process and a reverse denoising process. In the forward process, Gaussian noise is progressively added to the initial embedding ${h_0}$ over multiple Markov steps to generate noisy samples $h_{1}^{{(i)}},h_{2}^{{(i)}},...,h_{t}^{{(i)}}$. This process follows the conditional distribution, as shown in Eq. (9):

9 $${q}{\mathrm{(}}{h}_{t}^{{(i)}}\left| {{h}_{0}^{{(i)}}} \right.{\mathrm{)}}{=}{\mathrm{N(}}{h}_{t}^{{(i)}}{;}\sqrt {{{\bar {\alpha }}_t}} {h}_{0}^{{(i)}}{,}{\mathrm{(}}{1-}{\bar {\alpha }_t}{\mathrm{)}}{I}{\mathrm{),}}$$

where N denotes the Gaussian distribution, ${\bar {\alpha }_t}=\prod {_{{i=1}}^{t}} (1 - {{{\upbeta}}_{i}})$ controls the noise level at time step t, and I is the identity matrix. The closed-form expression for generating at any step t is:

10 $${h}_{t}^{{(i)}}{=}\sqrt {{{\bar {\alpha }}_{t}}} {h}_{0}^{{(i)}}{+}\sqrt {1 - {{\bar {\alpha }}_{t}}} \varepsilon ,{\text{ }}\varepsilon \in {\mathrm{(0,}}{I}{\mathrm{),}}$$

The reverse process aims to recover the original data $\hat {h}_{0}^{{(i)}}$ from the noisy observation $\hat {h}_{t}^{{(i)}}$ by learning a neural network ${\varepsilon _\theta }(\hat {h}_{t}^{{(v)}},t)$ that predicts the added noise. ${\varepsilon _\theta }$ is typically instantiated as a graph neural network that takes both $\hat {h}_{t}^{{(i)}}$ and the time step t as input, while incorporating structure-aware information from the auxiliary subgraphs. The denoising step is formulated as:

11 $${\hat {h}}_{{t - 1}}^{{(v)}}{=}\frac{{1}}{{\sqrt {{{\bar {\alpha }}_{t}}} }}\left( {{{{\hat {h}}}_{{t-}{\mathrm{1}}}}{-}\frac{{1 - {{\bar {\alpha }}_{t}}}}{{\sqrt {1 - {{\tilde {\alpha }}_{t}}} }}{\varepsilon _{{\uptheta}}}{\mathrm{(}}{\hat {h}}_{t}^{{(v)}}{,t}{\mathrm{)}}} \right){+}{{{\upsigma}}_{t}}{z,}$$

where ${\sigma _t}$ is a predefined variance, ${\tilde {\alpha }_{t}}=\prod {_{{s=1}}^{t}} {\bar {\alpha }_s}$ and $z\sim N(0,{\mathrm{I}})$. The model is trained to minimize the discrepancy between the predicted noise and true noise via the following diffusion loss,

12 $${\mathcal{\mathcal{L}}_{diff}}={E_{h_{0}^{{(v)}},\varepsilon ,t}}\left[ {{{\left\| {{\varepsilon _\theta }(\sqrt {{{\bar {\alpha }}_t}} \hat {h}_{0}^{{(v)}}+\sqrt {1 - {{\bar {\alpha }}_t}\varepsilon } ,t) - \varepsilon } \right\|}^2}} \right],$$

We employ a cross-view denoising mechanism that jointly utilizes information from two auxiliary subgraphs ${\mathcal{\mathcal{G}}^1}$ and ${\mathcal{\mathcal{G}}^2}$. During reverse diffusion, the shared denoising network ${\varepsilon _\theta }$ for view i is conditioned on both its own noisy embedding ${\hat {h}}_{{t - 1}}^{{({\mathrm{i}})}}$ and the structural features ${\hat {h}}_{t}^{{({\mathrm{j}})}}$ from the other view (j≠*i*):

13 $${\hat {h}}_{{t - 1}}^{{({\mathrm{i}})}}{=}\frac{{1}}{{\sqrt {{{\bar {\alpha }}_{t}}} }}\left( {{{{\hat {h}}}_{{t-}{\mathrm{1}}}}{-}\frac{{1 - {{\bar {\alpha }}_{t}}}}{{\sqrt {1 - {{\tilde {\alpha }}_{t}}} }}{\varepsilon _{{\uptheta}}}{\mathrm{(}}{\hat {h}}_{t}^{{({\mathrm{i}})}}{,t,\hat {h}}_{t}^{{({\mathrm{j}})}}{\mathrm{)}}} \right){+}{{{\upsigma}}_{t}}{z,}$$

This cross-conditioning enforces consistency between the two semantic views and mitigates view-specific noise. The final denoised patient embedding is obtained by averaging the outputs:

14 $$h_{v}^{{diff}}=\frac{1}{2}\left( {\hat {h}_{0}^{{(1)}}+\hat {h}_{0}^{{(2)}}} \right),$$

It is important to note that the denoising network ${\varepsilon _\theta }$ is shared across both auxiliary subgraphs. This parameter-sharing scheme not only reduces the total number of model parameters but, more importantly, it enables the learning of a generic, view-invariant denoising function that captures the underlying, noise-free patient representation common to both clinical perspectives (P-D-P and P-D-P-O-P). The denoising network ${\varepsilon _\theta }$ is implemented as a 3-layer GCN with hidden dimension 128. During the reverse process, we use the DDIM sampler for efficient inference.

Through this cross-view conditioning mechanism that integrates structural information from both auxiliary subgraphs, the module effectively captures complex relations among patients, drugs, and procedures for downstream disease diagnosis.

### Disease diagnosis

Upon obtaining patient embeddings, disease classification is performed to classify potential disease categories. This stage includes representation normalization, linear mapping, and label-smoothing–based supervised learning.

To mitigate scale inconsistencies and improve training stability, LayerNorm is applied to the fused embedding of $h_{v}^{{HHGAT}}$ and $h_{v}^{{diff}}$, as shown in Eq. (14):

15 $$h_{v}^{{final}}=LayerNorm(\lambda h_{v}^{{HHGAT}}+(1 - \lambda )h_{v}^{{diff}}),$$

where $\lambda$ is a fusion coefficient balancing structural semantics and noise robustness. LayerNorm ensures consistent distribution across embeddings, alleviating gradient instability. The normalized node representations are fed into a linear classifier to produce logits, which are then transformed into class probabilities via the Softmax function, as shown in Eq. (15):

16 $${{\hat {y}}_v}={Softmax}({W_c}h_{v}^{{final}}+{b_c}),$$

Here, ${W_c} \in {{R}^{{C}{{ \times }}{d}}}$ and ${b_c} \in {{R}^{C}}$ are the classifier parameters, and C is the number of disease classes. To enhance generalization, label smoothing transforms one-hot labels into smoothed distributions. Given a label ${y_v}$ and smoothing factor $\mu \in [0,1]$.With KL divergence minimized between prediction and smoothed label:

17 $${\mathcal{\mathcal{L}}_{{ClS}}}{=KLDiv}{\mathrm{(}}{log }{{\hat {y}}_v}\left\| {({1-}\mu ){y_v}{+}\frac{\mu }{{C}},} \right.{\mathrm{),}}$$

The total loss integrates classification and diffusion objectives:

18 $${{{\mathcal{L}}}_{\mathcal{total}}}={{{\mathcal{L}}}_{{cls}}}{+}{{{\mathcal{L}}}_{{diff}}}{,}$$

By combining hierarchical Transformer and diffusion-based representations, the model achieves both semantic expressiveness and robustness, enabling accurate and reliable multi-class disease diagnosis.

## Results

This section introduces the experimental datasets, evaluation metrics, baseline models, parameter settings, and results analysis. To comprehensively evaluate the proposed model, we conduct extensive experiments on the MIMIC dataset. Furthermore, we perform ablation studies, visualization, and hyper-parameter analysis to investigate the contribution of each model component.

### Datasets

This study utilizes the MIMIC-III and MIMIC-IV datasets, sourced from the ERs of Beth Israel Deaconess Medical Center, to construct medical heterogeneous graphs for disease diagnosis tasks, aiming to enhance model generalization and evaluation robustness.

**Table 1:** Dataset statistics (Zero entries indicate that the corresponding disease category is not included in that dataset.)

Disease label	MIMIC-III	MIMIC-IV
	#patients	#patients
Coronary disease	2750	0
Pneumonia	0	1256
Respiratory failure	388	0
Septicemia	0	2148
Disseminated infections	1830	0
Myocardial infarction	0	934
Heart failure	803	1629
Respiratory failure	1229	0
Diabetes	0	1557
Hypertension	0	807
Total	7000	8331

The disease labels used in MIMIC-III and MIMIC-IV differ, as summarized in Table 1, reflecting the distinct diagnostic focuses of each cohort. For this single-label classification task, only patients diagnosed with one of the predefined target diseases were retained, while others were excluded during dataset construction. Consequently, each patient in the final datasets is associated with exactly one disease label. In the MIMIC-III dataset, we select five representative diseases, including Coronary Disease, Disseminated Infections, Respiratory Failure, Heart Failure, and Gastritis. MIMIC-III comprises 7000 patients, 1379 types of medications, and 563 procedures. In the MIMIC-IV dataset, we choose six diseases: Septicemia, Heart Failure, Diabetes, Pneumonia, Myocardial Infarction, and Hypertension, involving a total of 8331 patients, 1692 medications, and 843 procedures.

The Table 2 provides a complete quantitative profile of the datasets. The high number of meta-path instances per patient, especially for P-D-P and P-D-P-O-P, indicates a densely connected semantic structure. This rich connectivity is leveraged by our k-hop sampling strategy to capture multi-scale clinical relationships. The sparsity of the primary patient-drug-procedure graph, measured as $1 - \left| {\mathrm{E}} \right|/\left| {\mathrm{V}} \right|$, is approximately 0.9875 for MIMIC-III and 0.9875 for MIMIC-IV, highlighting the extreme sparsity characteristic of real-world medical graphs.

**Table 2:** Structural statistics of patient–drug–procedure graphs in MIMIC-III and MIMIC-IV.

Coronary disease	MIMIC-III	MIMIC-IV
# Patient nodes (P)	7000	8331
# Drug nodes (D)	1379	1692
# Procedure nodes (O)	563	843
# P-D edges	137578	235097
# D-P edges	136201	233403
# P-O edges	33130	30036
# O-P edges	32571	29196
# P-D-P meta-path instances	47532615	68857653
# P-O-P meta-path instances	16666714	9063814
# P-D-P-O-P meta-path instances	48887343	68923786

### Evaluation metrics

Our task is formulated as a single-label disease classification, where each patient is assigned exactly one disease label. For this problem setting, we adopt Micro-F1 and Macro-F1 as the primary evaluation metrics, as they are widely recognized for medical classification tasks. Micro-F1 reflects the overall diagnostic accuracy by aggregating predictions across all disease categories, while Macro-F1 gives equal importance to each class, ensuring that rare diseases are adequately represented in the evaluation. Together, these metrics provide a balanced and interpretable assessment of model performance. The detailed formulations of Micro-F1 and Macro-F1 are shown below.

Micro-F1 aggregates predictions across all classes into a single evaluation, effectively reflecting overall performance and robustness to class imbalance:

19 $$Micro-F1=\frac{{2 \times TP}}{{2 \times TP+FP+FN}},$$

Macro-F1 computes the F1-score separately for each class and averages these scores, thus highlighting the performance across individual classes, especially those with fewer samples:

20 $$Macro-F1=\frac{1}{n}\sum\limits_{{i=1}}^{n} {\frac{{2 \times T{P_i}}}{{2 \times T{P_i}+F{P_i}+F{N_i}}}} ,$$

Here, TP denotes true positives, FP false positives, FN false negatives, and n the number of classes.

### Baseline methods

In this section, we briefly introduce several representative baseline methods used to evaluate the performance of our proposed TD4DD:

- GCN^27^ performs spectral convolution over graph structures by aggregating neighbor features based on Laplacian matrix transformations.
- GAT^23^ leverages self-attention to dynamically weight neighboring nodes and employs multi-head attention to enhance representation capacity.
- HAN incorporates hierarchical attention at both node and semantic levels, guided by predefined meta-paths to capture heterogeneous semantics.
- GIN utilizes sum-based neighbor aggregation followed by MLPs, resulting in node embeddings that are highly distinguishable across different classes.
- FastGTN learns high-order adjacency matrices through automatic graph transformation, enabling effective modeling of long-range dependencies.
- HGT adopts type-specific projections and multi-head attention to model heterogeneity in both node and edge types.
- HHGT introduces hierarchical attention mechanisms to differentiate neighbor semantics across varying hop distances in heterogeneous graphs.
- SlotGAT proposes a slot-assignment strategy to preserve type-aware representations in heterogeneous settings.
- DiffGraph^26^ integrates diffusion-based denoising mechanisms to refine noisy auxiliary information, yielding task-specific semantic embeddings.
- HeCo^13^ introduces a cross-view contrastive learning framework that aligns representations between network schema and meta-path views, enhancing the consistency of heterogeneous node embeddings across relational perspectives.
- MAGNN^14^ performs intra- and inter-metapath aggregation by encoding intermediate nodes along each meta-path, effectively capturing rich semantic dependencies and improving metapath-level interpretability.
- BEHRT^16^ applies the Transformer architecture to longitudinal EHR sequences, modeling temporal dependencies among medical codes and enabling contextualized patient representations for clinical prediction tasks.
- HSGNN^12^ constructs similarity subgraphs guided by meta-paths and fuses them with learnable weights, achieving hierarchical information integration.

### Parameter settings

For our TD4DD model we employ the Adam optimizer with an initial learning rate of 0.003 and train for up to 300 epochs. We use node embeddings of dimension 128 and three graph convolutional layers. The k-hop and hierarchical Transformer modules both use 4 attention heads. We apply label smoothing with a smoothing factor of 0.3. In the diffusion module embeddings are normalized and Gaussian noise of scale 1e-5 is injected during the diffusion process. All models are evaluated on a 50% training, 30% validation and 20% test split under identical computational conditions to ensure fair comparison. This partition ensures ample training data while offering a reliable assessment of generalization.

### Experimental results analysis

Table 3 shows that traditional graph convolution methods such as GCN and GIN fall short when modeling the complex interactions among patients, medications and procedures. Attention-based models such as GAT partially address this by assigning learnable weights to neighbors, but they still struggle with heterogeneous semantics. Heterogeneous graph techniques, for example HGT with type-specific attention and SlotGAT with slot-based feature separation, deliver further gains yet remain limited in capturing dynamic clinical relationships and multi-scale dependencies.

In contrast, TD4DD consistently outperforms all baselines on both the MIMIC-III and MIMIC-IV datasets, confirming its robustness in disease diagnosis over complex medical graphs. On MIMIC-III it achieves a micro-F1 score of 88.29 and a macro-F1 score of 86.11, while on MIMIC-IV it records a micro-F1 of 83.60 and a macro-F1 of 83.94.

**Table 3:** Performance comparison of different methods.

Methods	MIMIC-III		MIMIC-IV	
	Micro-F1	Macro-F1	Micro-F1	Macro-F1
GCN	84.76	80.31	80.16	80.47
GAT	86.43	84.21	82.16	82.86
HAN	84.76	81.55	76.00	76.33
GIN	86.90	83.43	80.84	81.49
FastGTN	80.13	80.13	78.96	78.96
HGT	85.81	82.30	77.28	78.25
HHGT	85.09	81.88	78.64	79.26
SlotGAT	87.62	85.22	82.12	82.49
DiffGraph	87.27	84.93	82.30	82.98
HeCo	86.12	83.47	81.25	81.83
MAGNN	86.45	83.40	82.13	82.06
BEHRT	82.20	80.10	78.50	78.90
HSGNN	86.20	82.60	81.52	81.90
TD4DD	88.29	86.11	83.60	83.94

### Ablation study

**Fig. 3:**
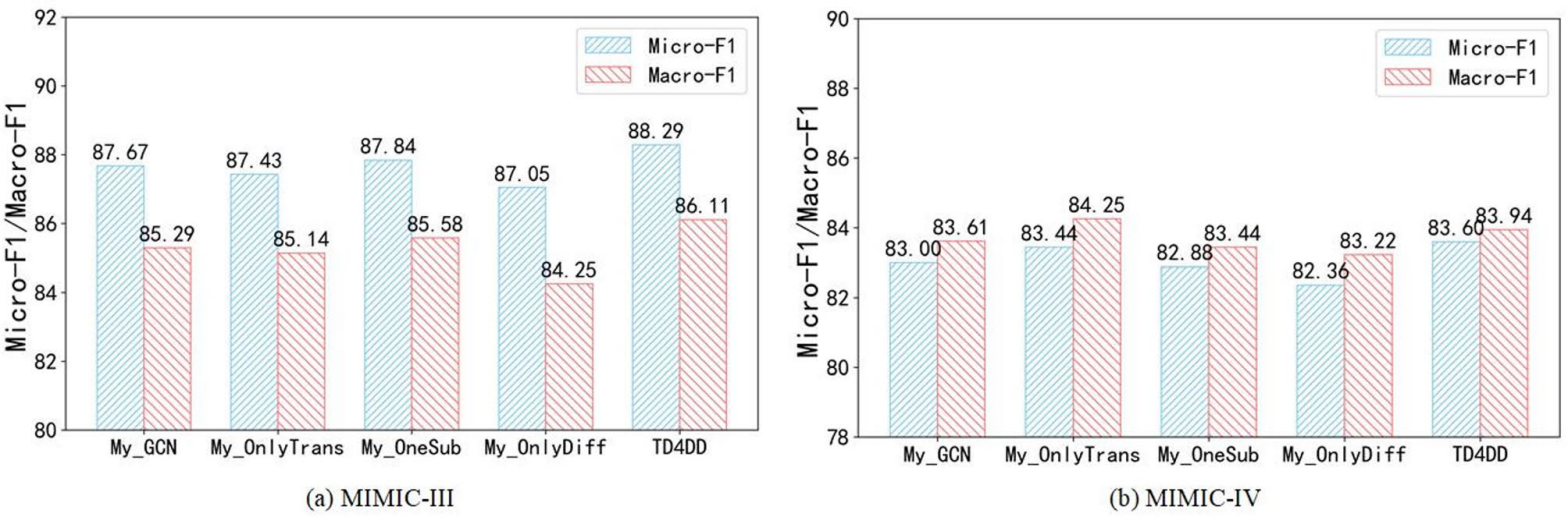
Ablation study of the TD4DD.

To quantify the contribution of each component in TD4DD, we create four ablated variants: My_GCN, My_OnlyTrans, My_OneSub, and My_OnlyDiff. The performance of these variants on both datasets is summarized in Fig. 3.

1. My_GCN: Replacing the hierarchical k-hop Transformer with a GCN lowers performance on both benchmarks; on MIMIC-IV the Micro-F1 drops to 83.00, demonstrating that the Transformer is indispensable for modeling long-range dependencies and multi-scale semantics.
2. My_OnlyTrans: Removing the entire diffusion module reduces the Micro-F1 on MIMIC-III from 88.29 to 87.84 and on MIMIC-IV from 83.60 to 83.44, underscoring the diffusion module’s critical contribution to denoising and enhancing representation robustness.
3. My_OneSub: only the P–D–P auxiliary view yields marginal gains over the complete removal of diffusion on MIMIC-III, yet the Micro-F1 on MIMIC-IV is limited to 82.88. This result confirms that a multi-view auxiliary structure is essential for maintaining semantic consistency.
4. My_OnlyDiff: Keeping only the diffusion module while removing the k-hop Transformer substantially degrades structural modeling capability, with the Micro-F1 dropping to 84.52 on MIMIC-III and 83.22 on MIMIC-IV, indicating that diffusion alone cannot capture hierarchical semantic dependencies.

In summary, the k-hop Transformer, diffusion module, and multi-view auxiliary subgraphs constitute the core of TD4DD; their synergistic interaction markedly strengthens the model’s robustness and discriminative power.

### T-SNE visualization

**Fig. 4:**
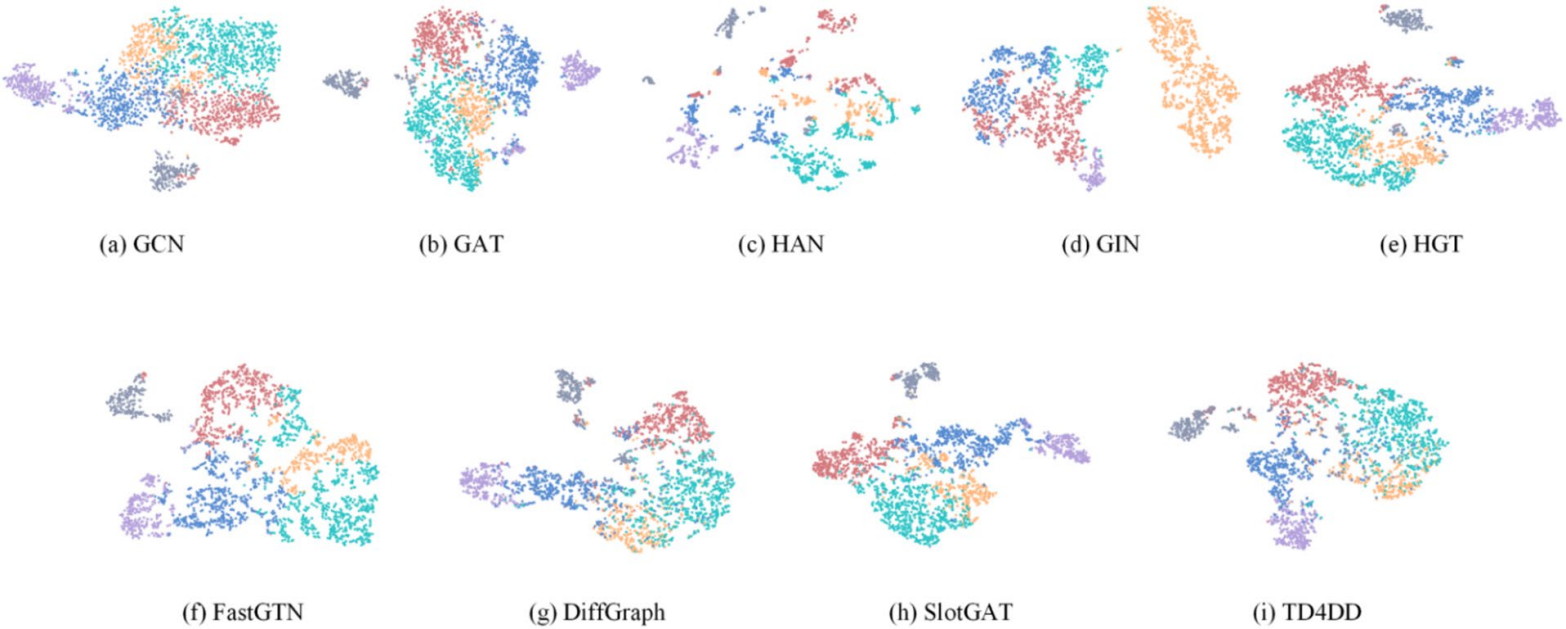
Visualization of patient node embeddings.

To visually evaluate the separability of the learned embeddings, we employ the t-distributed stochastic neighbor embedding t-SNE algorithm^30^ to project the final patient representations from MIMIC-IV into two dimensions. The visualization results are shown in Fig. 4, where different colors represent different types of disease labels.

The embeddings generated by GCN and GIN exhibit loose clustering with indistinct class boundaries. GAT and HAN show slight improvements in structural separation, yet label mixing persists. HGT and FastGTN produce more compact intra-class clusters but still exhibit overlap between classes. SlotGAT and DiffGraph further enhance class separability. In contrast, TD4DD achieves the most compact intra-class distributions and the clearest inter-class boundaries, demonstrating its superior ability to preserve high-order semantics and resist clinical noise.

### Hyperparameter analysis

To identify optimal settings, we vary one hyper-parameter at a time while keeping the others fixed.

1. Node Feature Embedding Dimension

The embedding dimension is critical for both the representational capacity and generalization ability of the model. We test embedding dimensions of 16, 32, 64, 128, and 256.

As shown in Table 4, performance improves as the dimension increases up to 128, at which point the model achieves optimal Micro-F1 and Macro-F1 on both MIMIC-III and MIMIC-IV. Further increase to 256 leads to performance degradation, likely due to overfitting and noise accumulation. Therefore, an embedding dimension of 128 is selected as the default configuration for subsequent experiments.

**Table 4:** Impact of embedding dimension on model performance.

Dim	MIMIC-III		MIMIC-IV	
	Micro-F1	Macro-F1	Micro-F1	Macro-F1
16	83.00	79.77	77.96	78.88
32	84.43	81.46	79.12	80.13
64	87.71	85.22	81.88	82.11
128	88.29	86.11	83.60	83.94
256	87.95	85.55	82.56	83.21

2. Label-smoothing coefficien

The label smoothing coefficient influences model regularization and classification robustness. We evaluate coefficients of 0.1, 0.3, 0.5, 0.7, and 0.9.

**Table 5:** Impact of label smoothing on model performance.

Label-smoothing coefficient	MIMIC-III		MIMIC-IV	
	Micro-F1	Macro-F1	Micro-F1	Macro-F1
0.1	87.57	85.18	82.84	83.58
0.3	88.29	86.11	83.60	83.94
0.5	87.90	85.68	83.24	83.95
0.7	87.76	85.57	82.88	83.75
0.9	39.29	11.28	18.58	1.33

As shown in Table 5, a coefficient of 0.3 yields the highest accuracy. Lower values provide little regularization, while higher values—especially 0.9—sharply reduce performance due to excessive blurring of class boundaries. Thus, 0.3 is selected as the optimal label smoothing coefficient.

3. Diffusion Noise Strength

Noise strength in the diffusion module determines the balance between denoising capability and information preservation. We teste values of 1e−6, 5e−6, 1e−5, 5e−5, and 1e−4.

**Table 6:** Impact of noise intensity on model performance.

Diffusion noise strength	MIMIC-III		MIMIC-IV	
	Micro-F1	Macro-F1	Micro-F1	Micro-F1
1e−6	88.14	85.88	83.48	84.06
5e−6	87.90	85.68	83.28	84.08
1e−5	88.29	86.11	83.60	83.94
5e−5	87.95	85.62	83.20	83.68
1e−4	87.95	85.80	83.16	83.68

As shown in Table 6, the best performance is obtained when the noise strength is set to 1e−5. Smaller values are insufficient for robust denoising, while larger values can corrupt semantic information and reduce accuracy. Therefore, a noise strength of 1e−5 is used in all reported experiments.

Overall, the configuration with embedding dimension 128, four attention heads, label smoothing coefficient 0.3, and diffusion noise strength 1e−5 achieves stable and optimal results on both datasets.

4. Impact of K-hop

The hop number k in the hierarchical Transformer directly controls the range of information propagation in the heterogeneous graph and determines how much contextual knowledge is integrated into each node representation. We evaluated k values from 1 to 5 to assess its effect on diagnostic performance.

As summarized in Table 7, model performance on both MIMIC-III and MIMIC-IV improves consistently as k increases from 1 to 4, indicating that incorporating higher-order neighborhood information enriches the clinical semantics learned by the model. The best results are achieved at k = 4, with Micro-F1 scores of 88.29 and 83.60 on MIMIC-III and MIMIC-IV, respectively. Increasing the hop number beyond this point leads to a noticeable decline, likely due to the introduction of noisy or less relevant information from distant neighbors, as well as potential over-smoothing effects.

**Table 7:** Impact of K-hop on model performance.

K-hop	MIMIC-III		MIMIC-IV	
	Micro-F1	Macro-F1	Micro-F1	Micro-F1
K = 1	87.47	85.22	82.98	83.64
K = 2	87.81	85.43	83.12	83.81
K = 3	87.85	85.65	83.16	83.86
K = 4	88.29	86.11	83.60	83.94
K = 5	87.42	85.12	83.16	83.76

Therefore, k = 4 is adopted in all experiments, as it provides the optimal balance between semantic richness and representation stability.

### Computational complexity and resources

To address practical deployment concerns, we report the computational resources required by TD4DD. The largest adjacency matrix constructed, corresponding to the P-D-P meta-path subgraph. For MIMIC-IV (|V|=8,331), this results in a matrix of approximately 69 million non-zero entries (as shown in Table 1).

The computational complexity of TD4DD is primarily governed by two components: the hierarchical Transformer and the diffusion module.

The self-attention mechanism in Transformer layers has O(N²·d) complexity for sequence length N and embedding dimension d. However, our k-hop sampling strategy limits the practical sequence length by processing only sampled neighborhoods per node, resulting in O(|V|·S²·d) complexity where S is the sampling size (32 neighbors per hop). The diffusion module employs a shallow GCN with O(|E|) complexity. Empirical measurements validate this analysis: on MIMIC-III with 7000 patients, training requires 16.94s per epoch and an average memory footprint of 10.3GB GPU memory, while on the larger MIMIC-IV (8,331 patients) it requires 29.58s per epoch and an average memory footprint of 14.4GB GPU memory on an NVIDIA T4(16GB), demonstrating practical scalability for clinical heterogeneous graphs of varying sizes.

## Conclusion

In this work, we propose TD4DD, a heterogeneous graph-based disease diagnosis framework that integrates a k-hop hierarchical Transformer with a diffusion mechanism to address key challenges in medical graph learning, including semantic confusion, type information loss, and noise interference. By leveraging meta-path-based structural inputs and auxiliary subgraphs, the model captures multi-scale semantic dependencies through hierarchical attention while performing cross-view denoising in latent space to enhance the robustness and distinguishing capability of node representations. Experimental results demonstrate that TD4DD consistently outperforms state-of-the-art baselines such as SlotGAT, FastGTN, HHGT, and DiffGraph, particularly in complex and imbalanced medical graph scenarios. Specifically, TD4DD achieves a Micro-F1 of 88.29 and a Macro-F1 of 86.11 on the MIMIC-III dataset, and obtains 83.60 and 83.94, respectively, on the MIMIC-IV dataset. Compared with the best-performing baseline SlotGAT, TD4DD improves Micro-F1 and Macro-F1 by 0.67 and 0.89 on MIMIC-III, and by 1.48 and 1.45 on MIMIC-IV.Moreover, ablation studies confirm the critical contributions of the diffusion module, auxiliary subgraphs, and Transformer architecture, while t-SNE visualizations further validate the model’s ability to learn high-quality and well-separated embeddings.

The experimental results confirm the effectiveness of TD4DD. From an interpretability standpoint, the model’s internal mechanisms align well with clinical reasoning. For instance, the learned attention weights in the hierarchical Transformer allow us to trace which neighbors and which hop levels contributed most to a prediction, potentially highlighting key medications or symptomatic relationships. The diffusion module’s denoising process inherently strengthens robust clinical patterns. These properties make TD4DD not only effective but also transparent and trustworthy for real-world clinical decision support.

## Data Availability

The data that support the findings of this study are derived from the Medical Information Mart for Intensive Care (MIMIC-III and MIMIC-IV) databases. These databases are publicly available to researchers who meet the necessary criteria for ethical and responsible data use. Researchers can apply for access through the official website: https://mimic.mit.edu/.
